# Integrated optical frequency comb for 5G NR Xhauls

**DOI:** 10.1038/s41598-022-20553-5

**Published:** 2022-09-30

**Authors:** Eduardo Saia Lima, Ramon Maia Borges, Nicola Andriolli, Evandro Conforti, Giampiero Contestabile, Arismar Cerqueira Sodré

**Affiliations:** 1 Laboratório WOCA, (Inatel), Santa Rita do Sapucaí, Brazil; 2 CNR-IEIIT, Via Caruso 16, 56122 Pisa, Italy; 3 DECOM-University of Campinas, Campinas, Brazil; 4 Scuola Superiore Sant’Anna, Via Moruzzi 1, 56124 Pisa, Italy; 5 Institute of Systems Engineering and Information Technology, Federal University of Itajubá (UNIFEI), Itajubá, Brazil

**Keywords:** Fibre optics and optical communications, Integrated optics

## Abstract

We experimentally demonstrate the use of optical frequency combs (OFCs), generated by a photonic integrated circuit (PIC), in a flexible optical distribution network based on fiber-optics and free-space optics (FSOs) links, aimed at the fifth generation of mobile network (5G) Xhauls. The Indium Phosphide (InP) monolithically integrated OFC is based on cascaded optical modulators and is broadly tunable in terms of operating wavelength and frequency spacing. Particularly, our approach relies on applying the PIC in a centralized radio access network (C-RAN) architecture, with the purpose of optically generating two low-phase noise mm-waves signals for simultaneously enabling a 12.5-km of single-mode fiber (SMF) fronthaul and a 12.5-km SMF midhaul, followed by a 10-m long FSO fronthaul link. Moreover, the demonstrator contemplates two 10-m reach 5G wireless access networks operating in the 26 GHz band, i.e. over the frequency range 2 (FR2) from the 5G NR standard. The proposed integrated OFC-based 5G system performance is in accordance to the 3rd Generation Partnership Project (3GPP) Release 15 requirements, achieving a total wireless throughput of 900 Mbit/s.

## Introduction

Over the years, wireless connectivity has driven major societal changes, which are expected to be significantly intensified with the fifth-generation of mobile network (5G). Such generation enables an ecosystem of new value-added applications, which started to be deployed mainly focusing on the enhanced mobile broadband, while other scenarios continue acquiring maturity. Remarkable 5G technical solutions include^1–3^: the definition of the 5G New Radio (5G NR) standard; the use of spectrum in the millimeter-wave (mm-wave) band; the employment of heterogeneous and centralized radio access network (C-RAN); the optical-wireless convergence. In parallel, the sixth-generation of mobile network (6G) has become a hot topic, planned to address possible unfulfilled promises from 5G, providing superior efficiency and satisfying future demands from 2030 and beyond. Among the potential technical solutions for 6G^4,5^, one can highlight the use of THz waves, as well as the use of artificial intelligence/machine learning to support autonomous networks, and an innovative air-interface design.

Indeed, both 5G and 6G indicate trends on higher operating frequencies and broader bandwidths (BWs). 5G requires mm-wave hotspots, which shall be implemented in the 5G NR frequency range two (FR2) from 24.2 to 52.6 GHz^1^. 6G prospects point out to even higher frequencies for reaching beyond Gbit/s throughput in the wireless environment^5^. However, it is well known that generating and distributing mm-waves brings complexity and represent challenging tasks, especially when working only in the electrical domain. In this context, microwave photonics (MWP) plays an important role in supporting the new wireless generations, by means of enabling the desired optical-wireless convergence and promoting attractive solutions for mm-waves distribution and generation^6,7^.

**Figure 1:**
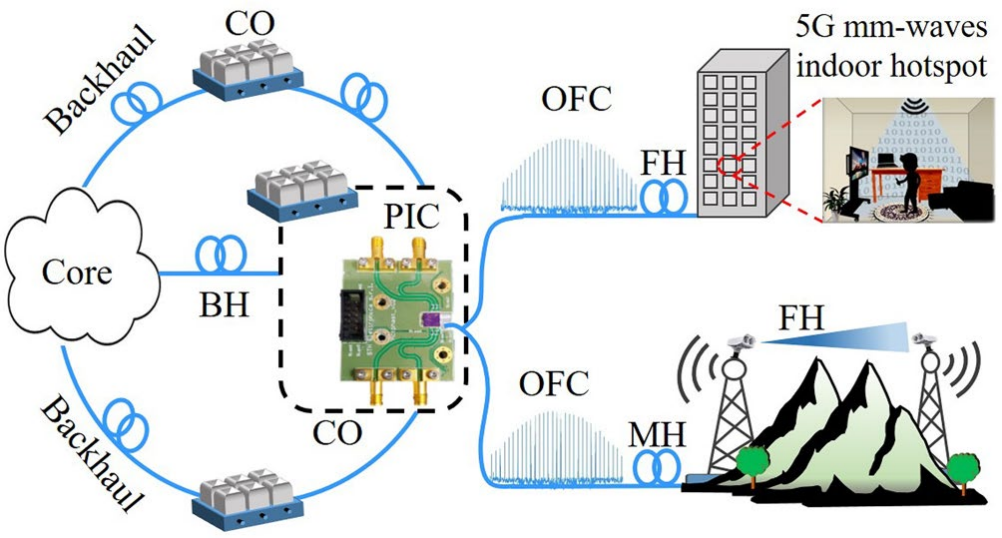
The proposed 5G NR system based on an integrated OFC. *BH* backhaul, *CO* central-office, *FH* fronthaul, *MH* midhaul, *FSO* free-space optics, *OFC* optical frequency comb, *PIC* photonic integrated circuit.

The transport of mm-waves signals over fiber-optics links is efficiently ensured by using radio over fiber (RoF) technology, which might be digital RoF (D-RoF), analog RoF (A-RoF), or hybrid^8–10^. D-RoF schemes digitize analog signals for launching them into the optical link, whereas A-RoF schemes transmit radiofrequency (RF) signals in nature through the fiber optics, i.e., the distribution occurs already in the channel frequency. A hybrid solution combines D-RoF and A-RoF in order to merge the advantages of both techniques. Alternatively, free-space optics (FSO) technology has also shown potential for the mm-waves transport network^11–13^. FSO links have been demonstrated in conjunction with RF links and combined with RoF, in which the benefit is enabling a dual link and/or last-mile applications when there are restrictions on fiber-optics link installation. The system flexibility is then increased, as a consequence of hybrid RoF/FSO approaches^3^. It is worth highlighting that RoF and FSO have been recognized as promising to deploy optical fronthauls, which is a link connecting the central office to the remote mm-waves radio units in the so-called “Xhaul” architecture^14,15^, as presented in Fig. 1.

The photonics-assisted mm-waves generation has been mainly demonstrated using three methodologies. The first one is optical heterodyne, which exploits the beating of two optical waves at the photodetector (PD), resulting in an electrical signal with a frequency equal to the difference between the two original waves^7^. This strategy, although simple, suffers from prohibitive phase noise, due to the absence of correlation of the light sources. For this reason, complex techniques, such as dual-wavelength mode-locked laser (MLL)^16^, optical injection locking^17^, and optical phase-lock loop^18^ are required to enable low phase noise mm-wave generation using the heterodyne approach. The second highlighting methodology refers to the optical frequency multiplication (OFM), commonly based on external modulation techniques^19–21^. This strategy is based on a frequency multiplication factor, e.g. 2, 4, 6, 8 or 12 depending on the setup applied, for allowing RF upconversion to microwaves or mm-waves. Spectral purity and non-limiting phase noise are typically achieved with OFM, however, the number of cascaded elements increases as a function of the multiplication factor^21^. The third potential methodology for generating mm-waves deals with OFC, which enables to accurately transfer information from a stable spectral component (a reference) to many tones in the optical domain^22^. The latter ones can be used to generate mm-wave spectral components in the electrical domain after the photodetection. Different techniques have been used for OFC generation, including MLL^22^, multiple four-wave mixing^23^, and electro-optics modulators^24^. It is worth highlighting that microwaves and mm-waves generation with attractive performance in terms of phase noise have been achieved by taking advantage of OFCs^25–29^.

**Table 1:** Photonics-based frequency generation and multiplication.

References	Frequency (GHz)	Technique	Based on PIC	OFM	Optical link (km)	Goal/accomplishment
^19^	6	External modulation	No	2	1.5	Mobile network/yes
^20^	40	External modulation	No	4	50	RoF systems/no
^21^	100	External modulation	No	4, 6 and 8	–	Not applicable
^17^	101.7	OIL	Yes	–	–	Not applicable
^18^	45.4	OPLL	No	–	–	Not applicable
^27^	94.8	SOFCG	No	8	2	Mobile network/yes
^28^	36	Kerr combs	No	Up to 3x	No	Not applicable
^29^	72	E/O OFC/MLL	No	Up to 12x	–	Not applicable
^25^	10/20	Soliton microcombs	Yes	2	–	Radar/yes
This work	26	E/O OFC	Yes	10	12.5	5G mm-waves/yes

Recent progress on IMWP^30^, a segment that focuses on combining optical components in a single chip, has indicated a breakthrough in OFC-based mm-wave generation and high-capacity optical communications. The acquired benefits include: moving from several components on bench-top to footprint-reduced chips; operation simplicity; scalability; versatile features for specific types of comb and design parameters; energy saving; tight confinement of light due to the high index contrast, increasing optical nonlinearities, favoring to perform and phase match parametric nonlinear processes^31,32^. Then, works on integrated OFCs at the device level have been reported as state-of-the-art in the literature^25,31–36^. For instance, Hu and Oxenlowe presented a review on chip-based OFCs, in which different types of comb benefiting from silicon, thin-film lithium-niobate, and aluminium gallium arsenide-on-insulator were discussed, as a function of optical linewidth, spectral broadening and carrier-to-noise ratio^33^. Aiming for ultra-dense optical transmission, Corcoran et al. exploited a soliton crystal comb using a fiber-optic packaged micro-ring resonator chip with 5 x 9 mm^2^ in size^34^. More than 40 Tb/s over 76.6 km of SMF was demonstrated by employing a single chip source.

Table 1 presents several works focused on photonics-based frequency generation and multiplication. Regarding the techniques based on external modulation, Borges et al. in^19^ reported a radio frequency converter, the proposed optoelectronic device provided photonics-based up-conversion and down-conversion depending on the applied bias voltage. Experimental results demonstrate 1.5-km optical distribution and radiofrequency doubling over the frequency range from 750 MHz to 6.0 GHz. In^20^, Lin et al. provided a theoretical analysis and experimental demonstration of a photonics-based frequency quadrupling. The authors employed only one external modulator without optical filtering to generate a 40 GHz electrical carrier with high-spectral purity. On the same token, the authors in^21^ reported a theoretical analysis followed by an experimental validation regarding the use of two cascaded MZM to achieve frequency quadrupling, sextupling, or octupling based on the MZM operating point, achieving 100 GHz. In the context of locking the phase of optical carriers, Balakier et al. in^17^ employed two monolithically integrated semiconductor lasers to generate mm-waves. Carriers up to 101.7 GHz with relatively high-spectral purity have been achieved by optically injection locking the lasers to a common optical frequency comb used as a reference. The authors in^18^ reported a dual-loop optical feedback for stabilizing the frequency fluctuations and photonically generating a 45.424 GHz signal with a linewidth below 50 kHz.

Regarding optical frequency combs for frequency generation and multiplication, a self-oscillating optical frequency comb generator (SOFCG) has been demonstrated by applying an optoelectronic loop feedback to an OFC generator based on MZM^27^. The SOFCG carrier spacing has been set to 11.84 GHz, sequentially, two tones spaced by 8 times the repetition rate have been selected and detected by a high-speed photodetector. Finally, a 2-km RoF link followed by 1.3-m wireless link at 94.8 GHz has been demonstrated by transmitting a 20-MHz bandwidth signal. Additionally, a Kerr OFC has been applied to provide mm-waves generation and multiplication up to 3 times, achieving 36 GHz^28^. In^29^ authors employed an E/O frequency comb in conjunction with an MLL aiming to enhance the phase noise of mm-waves signals provided by conventional signal generators. Phase noise reduction in an arbitrary frequency between 6 and 72 GHz has been reported assisted by E/O frequency combs. Liu et al. demonstrated the use of an integrated soliton micro combs operating at the K- and X-bands. The authors reported the generation of low-phase noise signals at 10 and 20 GHz, and its experimental application in a radar system^25^.

In this paper, we experimentally demonstrate the use of an integrated OFC to generate mm-waves 5G NR signals, as illustrated in Fig. 1. Our approach encompasses an electrical band-pass filter used for isolating the low-phase noise 26 GHz carrier from the 2.6-GHz spaced electrical frequency comb, enhancing the system spectral efficiency. To the best of our knowledge, for the first time, a photonic integrated OFC is applied to a real 5G mm-waves access network. The PIC is physically located at the central office (CO) and diverse optical frequency combs are generated by splitting the original one, aiming for multiple OFC applications using a single PIC. The OFC distribution occurs via fiber-optics and in a hybrid approach, 12.5 km of SMF followed by a 10-m reach FSO link, enhancing the system flexibility, specially for places where the optical fiber use is impractical. In other words, our approach relies in applying the PIC in a centralized radio access network (C-RAN) architecture, with the purpose of optically generating two low-phase noise mm-waves signals for simultaneously enabling a 12.5-km SMF fronthaul link and a 12.5-km SMF midhaul, followed by a 10-m long FSO link acting as a fronthaul. Furthermore, our 5G NR system comprises two 10-m reach wireless access networks operating in the 26 GHz band, i.e., over the frequency range (FR2) from the 5G NR standard. The proposed integrated OFC-based 5G system performance is evaluated as a function of the 3rd Generation Partnership Project (3GPP) Release 15 requirements.

Comparing this work with the main papers in the literature, just a few works have reported photonic integrated circuits or optical frequency combs applied to mm-waves generation. In addition, several works reported the photonics-assisted generation, however, a minor part focuses on the optical fronthaul link distribution to provide a remote generation, neglecting the chromatic dispersion (CD) impairment. Finally, the majority of the papers only focused on the photonic generation and do not indeed apply the generated low-phase noise mm-wave carrier. Therefore, this work stands out in respect of a full system implementation, encompassing the mm-waves generation based on integrated optics, hybrid optical link distribution based on fiber-optics and FSO links, and the practical deployment of 5G NR hotspots operating at 26 GHz based on the integrated OFC.

## Integrated OFC-based 5G NR system: methods/experiments

**Figure 2:**
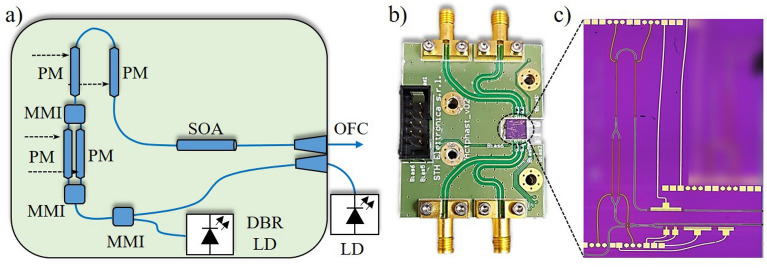
Indium phosphide monolithically integrated optical frequency comb based on cascaded modulators. (**a**) PIC schematic; (**b**) picture of the integrated OFC; (**c**) zoom-in-view of the chip on the metal chuck.

**Figure 3:**
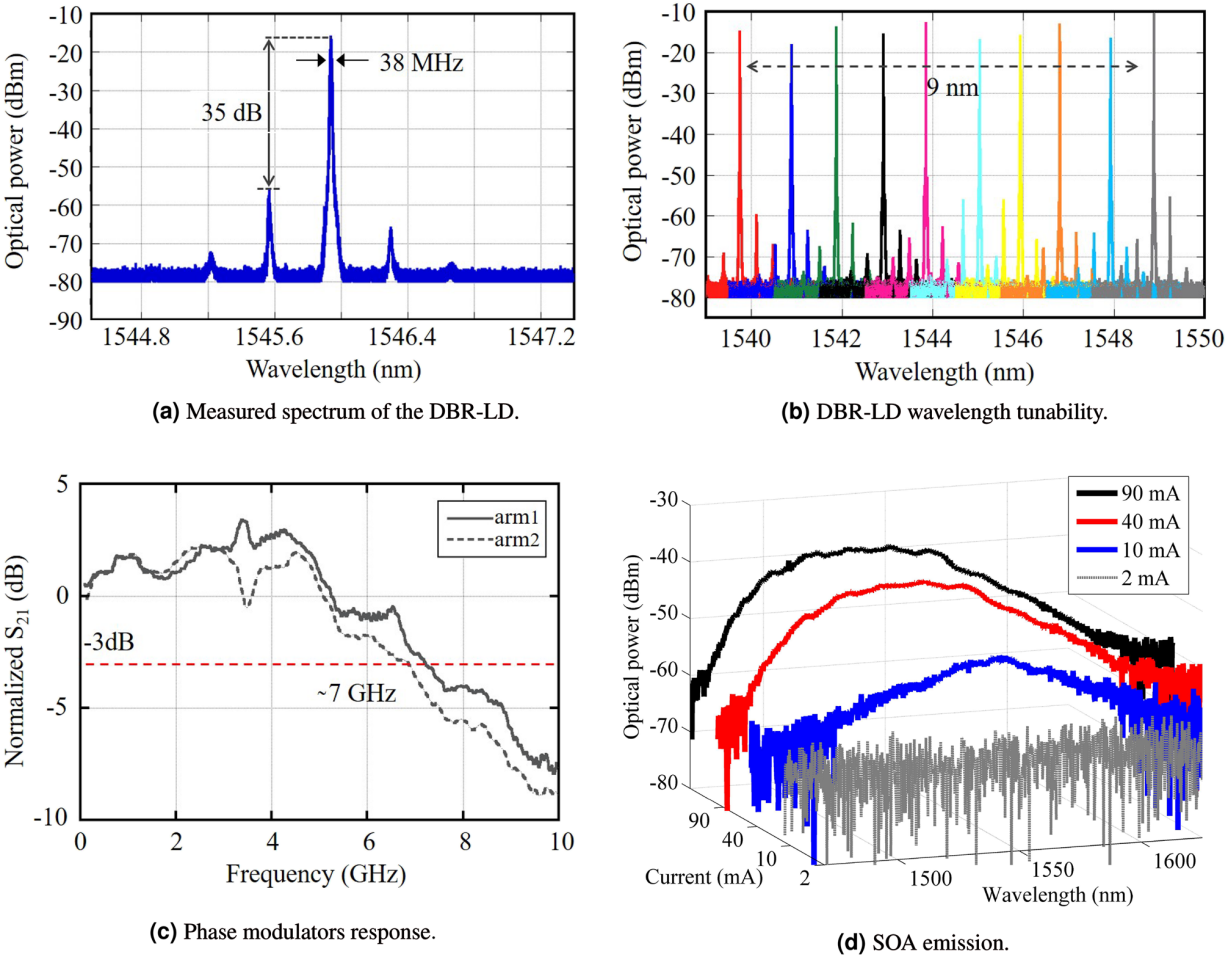
Characterization of the photonic integrated circuit.

The InP monolithically integrated optical frequency comb generator^35,36^ schematic, photography, and chip zoom-in-view are presented in Fig. 2a–c, respectively. The device has been fabricated in a InP multi-project wafer run at Oclaro Technology plc, UK. The PIC footprint is around 4.5 $\times$ 2.5 mm^2^, stipulated by the phase modulators and gain section placement. The on-chip integrated optical devices are connected via deeply-etched passive waveguides with 1.5-$\upmu$m width and 5-dB/cm loss. The chip was attached to a metal base employing a thermo-electric conductive epoxy and wire bonded to a custom-built PCB, as illustrated in Fig. 2b. Four RF SMA connectors have been used to drive the phase modulators, whereas 5 pins of a general purpose input/output (GPIO) have been used to supply and control the distributed DBR-LD sections and the SOA. A CW optical carrier, dictating the OFC central frequency, can be provided by an on-chip DBR-LD or using an external laser source. Basically, the integrated laser is composed of two DBR grating mirrors with a 237.5 nm pitch, a 450-$\upmu$m-long gain section and a 50-$\upmu$m-long phase tuning section, which enables wavelength tuning of the laser emission. Figure 3a,b present the integrated DBR-LD optical spectrum and the wavelength tunability, respectively. The LD threshold current occurs around 25 mA injected current. In addition, the DBR-LD has a monochromatic emission with a side-mode suppression ratio (SMSR) of 35 dB, and 38 MHz linewidth directly measured by a heterodyne optical spectrum analyzer^35^. Regarding the DBR-LD wavelength tuning, it can be achieved by setting a stable current in the LD gain section and varying the current of the remaining three sections, i.e., LD back mirror, front mirror, and phase control. One can note 9 nm of tunability with a 4 dB variation from the maximum emission. In addition, the operating wavelengths are distinct for the various fabricated chips, ranging between 1539 and 1558 nm.

Subsequently, the CW optical carrier is split using a 1 $\times$ 2 MMI reaching two 1-mm-long PMs associated in parallel, which operate as a DD-MZM, creating an amplitude modulation on the signal. Subsequently, the optical signal propagates through a series of two identical 1-mm-long-PMs, adding a phase modulation to the signal. In order to evaluate the performance of the PMs positioned in the MZM branches, we have used a test MZM identical to the one employed in the OFC. The frequency response of the PMs has been measured by individually applying the signal from a vector network analyzer (VNA) on each arm of the MZM. The output signal has been sent to a photodetector and evaluated by the network analyzer. Figure 3c shows the normalized transmission coefficient (S_21_) of the phase modulators positioned in the MZM branches. Both modulators present a 3 dB bandwidth of around 7 GHz. The BW is limited by the lumped electrical contacts and a slight 50 Ohm termination mismatch. Concerning the insertion loss of PMs, it is not an absolute value, depending on the voltage bias, wavelength, and orientation. As shown in^37^, at limited bias, the loss is around 3 dB, but for higher bias the electro-absorption effect becomes significant, causing around 20 dB loss at 1550 nm when the bias is − 8 V.

The modulated signal reaches then a 500-$\upmu$m-long multi quantum-well SOA used to adjust the output power. Figure 3d reports the SOA emission as a function of the polarization current. One may note no amplified spontaneous emission (ASE) when biasing the SOA with 2 mA and a transparency condition is obtained around 10 mA. For polarization currents higher than 40 mA the device operates properly, occupying a large part of the optics communications C band, more specifically, 62 nm of 3-dB bandwidth centered at 1535 nm. In addition, a gain saturation can be observed as a function of the current increment. The SOA output reaches a SSC that optimizes the OFC coupling into the output optical fiber. A high numerical aperture fiber array has been placed at the chip edge by using a 3D micro-positioner. Therefore, the fiber array must be positioned as close as possible to the SCC for optimizing the optical power coupled into the array, ensuring that it does not touch the chip edge, since it can damage the PIC. The output optical power was around − 2 dBm, measured by a wide-area detector, whereas the coupled power was around − 7 dBm, resulting in a coupling/alignment loss of around 5 dB. The photonic integrated circuit power consumption is lower than 0.5 W, considering the SOA and DBR-LD consumption. Regarding the RF generator used (PSG E8267D from Keysight), the typical power consumption is around 450 W. It is worth mentioning that no thermoelectric cooler (TEC) was required in this case, the device was fixed on a metal surface used as a heat-sink. The heat from the SOA and the DBR-LD does not significantly impact the phase modulators Vpi, and consequently, the comb generator response. Therefore, no instabilities due to the thermal crosstalk effects have been experienced during the experiments. In addition, the temperature of the laboratory environment was controlled and always kept around 19 °C.

**Figure 4:**
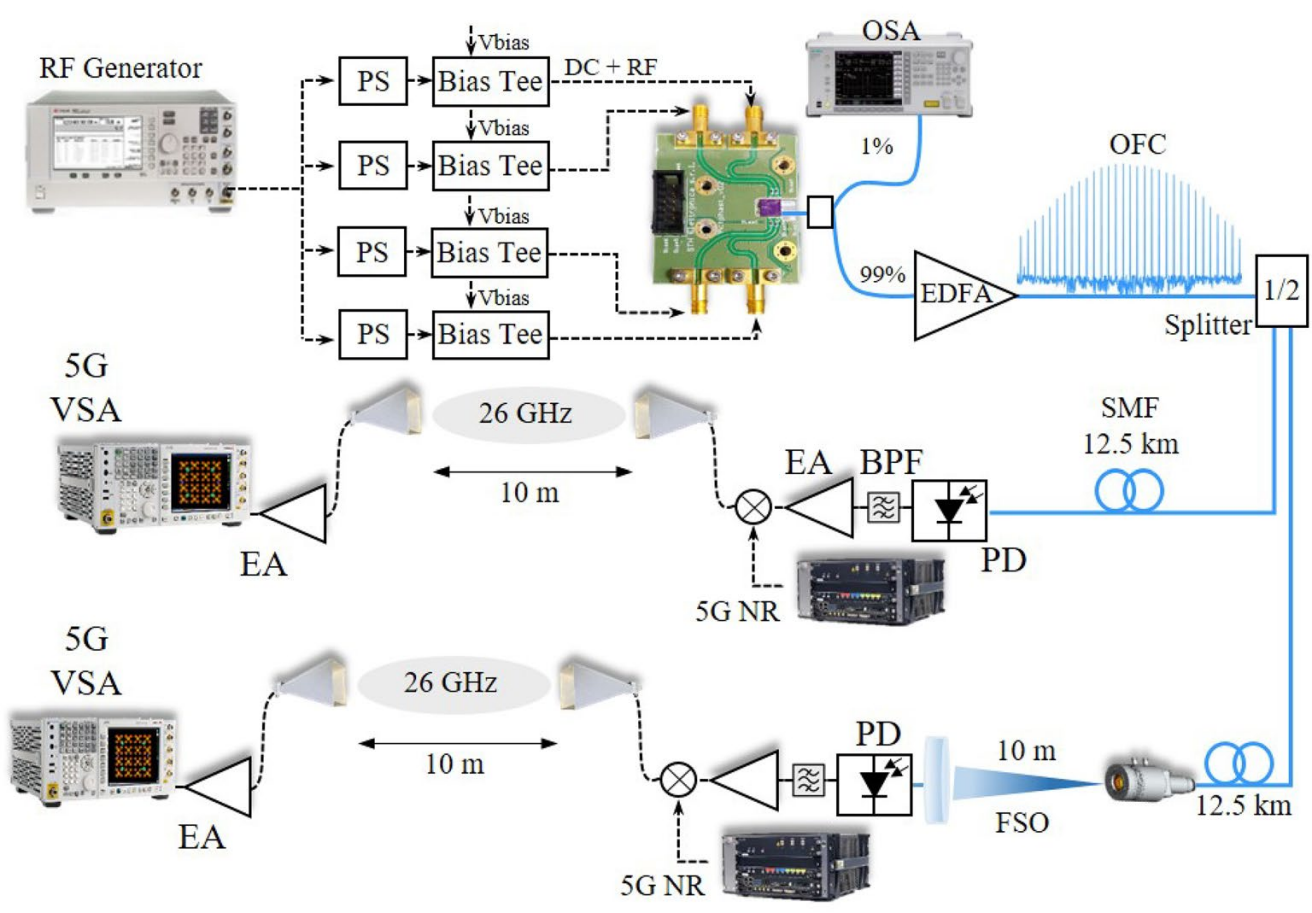
Experimental setup of the photonic integrated optical frequency comb-based 5G NR mm-waves system.

Figure 4 depicts a block diagram of the proposed OFC-based 5G system, which focuses on optically generating remote mm-waves signals, enabling the replacement of bulky and costly high-frequency oscillators at the remote radio units using a centralized integrated OFC. In order to generate the optical frequency comb, a PSG E8267D RF generator from Keysight has been used to create a 24.7-dBm electrical carrier at 2.6 GHz. This specific frequency has been chosen for producing a low-phase-noise mm-wave signal at 26 GHz, by taking advantage of the tenfold frequency multiplication provided by the OFC. High-frequency harmonics could also be used, for instance, 5.2 GHz. However, we focused on employing a relatively lower frequency as a comb source, reducing the complexity of the RF generator used, and in parallel, achieving a considerable frequency multiplication. Sequentially, a 1/4-electrical splitter has been used to equally divide the input signal, reaching four mechanically controllable RF PSs. The phase adjustment on each arm is important for optimizing the temporal phase alignment among the phase modulators, enhancing the OFC in terms of comb lines and spectral flatness^38^. Despite the electrical phase impact on the OFC, no critical stability issues coming from the RF phase shifters have been noticed, since slight phase variations do not significantly impact the OFC shape. Sequentially, bias tees have been utilized to couple the 2.6 GHz signal with the DC bias voltage before feeding the PIC PMs, using SMA connectors. The cascaded PMs have been driven with multiples of V$_\pi$, whereas the DD-MZM (PMs in parallel) with approximately V$_\pi$, aiming to enhance the OFC flatness and increase the number of comb lines^39^. As previously reported in^35^, the PM V$_\pi$ is around 5 V. Furthermore, the DBR laser and SOA current were 70 mA and 60 mA, respectively.

**Figure 5:**
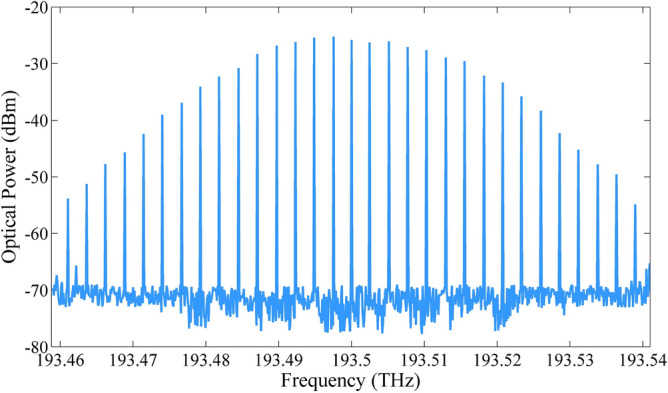
2.6-GHz optical frequency comb at the PIC output.

At the PIC output, the OFC is properly coupled into a high numerical aperture optical fiber, obtaining around − 7 dBm output optical power. In principle, higher optical power can be achieved, however, we have observed that setting higher optical powers in the SOA caused on-chip laser instabilities due to spurious reflections on the chip. Therefore, we operated the SOA output power guaranteeing a stable OFC operation. An unbalanced splitter with 1/99 split ratio has been used for monitoring an OFC sample (1%), whereas the remaining OFC power (99%) reaches an EDFA, which has been employed to compensate the optical link propagation losses. The EDFA output power was around 8 dBm (15 dB gain), ensuring 2-dBm optical power at the PD input. Figure 5 presents an example of the generated optical frequency comb centered at 193.5 THz with in-phase optical tones spaced by 2.6 GHz ranging from 193.46 to 193.54 THz. One can observe 17 optical carriers within a 10 dB intensity range from the maximum optical power and OSNR higher than 30 dB. In addition, 12 tones presented OSNR higher than 40 dB, which could be considered suitable for generating mm-waves signals over a wide frequency range. Sequentially, a 1 $\times$ 2 splitter, placed after the EDFA, has been used to equally divide the optical signal, creating two identical OFCs with approximately 4.5 dBm. In particular, we have used a 1 $\times$ 2 splitter as a proof-of-concept, splitters with higher division factors can be used to distribute the OFC to even more branches.

We have simultaneously employed the divided OFCs in two distinct 5G scenarios. The first one consisted of transmitting the OFC throughout a 12.5-km optical fronthaul, aiming to generate a low-phase-noise mm-waves signal at the remote radio unit (RRU). In this case, the received OFC power at the PD input was around 2 dBm. The generated carrier at 26-GHz was then modulated with the 5G NR standard and transmitted over a 10-m indoor wireless link with bandwidth up to 100 MHz. The second implemented scenario represents an evolution of the previous one since the 12.5-km SMF link acted as a midhaul and was followed by a 10-m reach FSO link, constituting a fronthaul. Finally, a 10-m wireless access link has been implemented as a 5G hotspot. In both cases, the OFC has been launched into a 12.5-km SMF with attenuation $\alpha$ = 0.2 dB/km and dispersion d $\approx$ 17 ps/nm$\cdot$km in the C band. In the hybrid fiber/FSO application, an optical collimator (Thorlabs F110APC-1550) with 6.3 mm focal length has been applied for the FSO link. It is worth mentioning that FSO systems must provide an eye-safe environment. Infrared communication in the 1.5 $\upmu$m band does not reach the retina at the same level of the visible light. However, optical powers higher than 10 dBm in conjunction with beam diameters lower than 1 cm may offer risks to the eyes^12^. For this reason, we have transmitted only 0 dBm and placed an EDFA at the reception side in order to deploy a safe free-space optics environment. At the receiver, we have positioned a condenser lens with a focal length of 200 mm and diameter of 75.0 mm for coupling the received OFC into an optical patch cord. The received beam diameter was lower than the lens diameter, as a consequence, the FSO losses were only the beam coupling/alignment to the fiber and TX/RX lenses efficiency.

**Figure 6:**
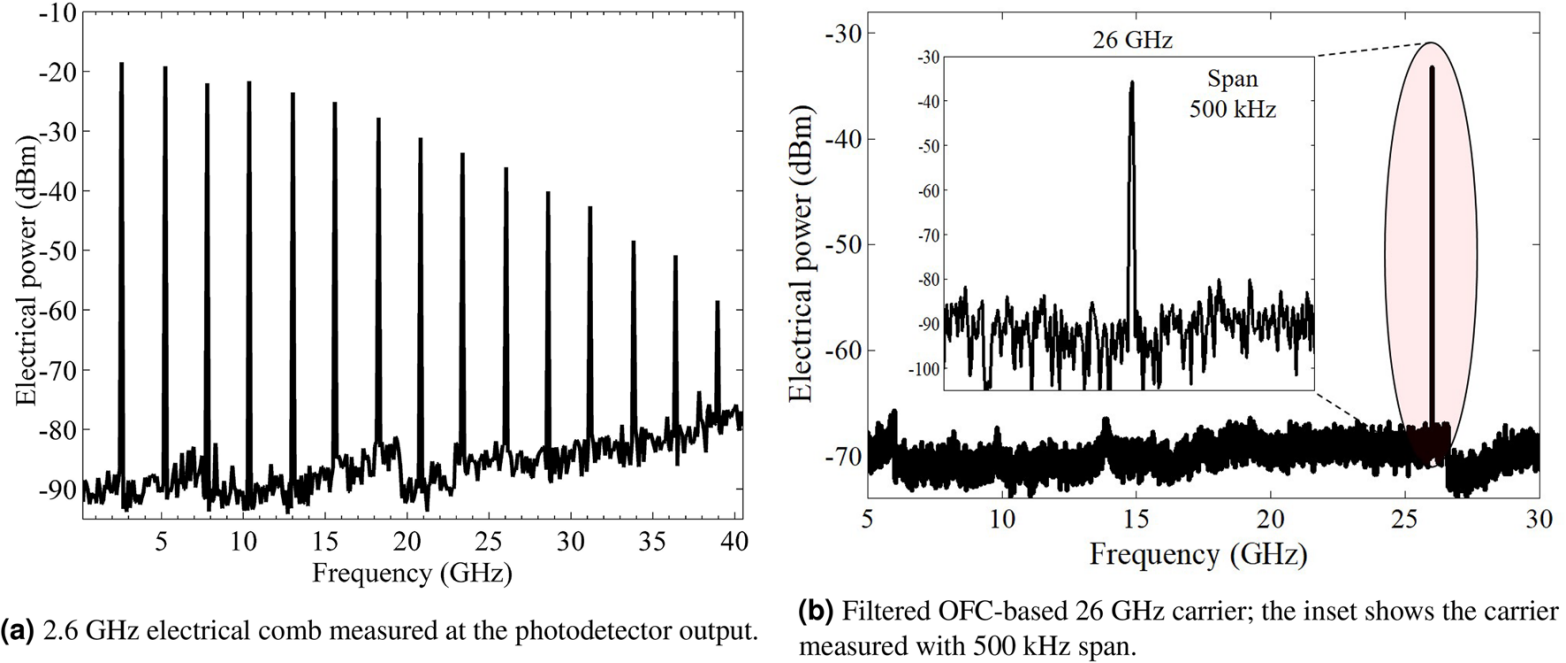
26-GHz signal generation using the integrated optical frequency comb.

The received optical power at the EDFA input was around − 8 dBm. The losses are mainly caused by the beam coupling into the SMF patch cord since a second collimator was not available on the reception side. Afterward, an EDFA has been used to overcome the FSO losses and enable a proper comparison of both scenarios operating at the same optical power, i.e, 2-dBm OFC power at the 74-GHz bandwidth photodetector (BPDV3120R) input. The 74-GHz PD was employed for research purposes, while in a practical application a 26 GHz photodetector would be sufficient. The in-phase optical lines have been converted to a set of equally spaced 2.6 GHz electrical tones and properly analyzed using an ESA (Anritsu MS2830A), as shown in Fig. 6a. One can observe a suitable electrical comb with 2.6 GHz harmonics ranging up to 41.6 GHz at a high SNR. For instance, SNR of 72 dB and 50 dB can be appreciated at 2.6 GHz and 26 GHz, respectively. In this way, the multiple generated carriers might be separated and applied to distinct applications, depending on the desired frequency range. Aiming to cover the entire FR2 (24.2–52.6 GHz) or even higher frequencies, our proposed approach is scalable in two ways. The first one consists of increasing the OFC frequency spacing up to 7 GHz, dictated by the phase modulators bandwidth, since we have used 2.6 GHz aiming to demonstrate a high OFM. A second way comprises the use of electrical drivers at the phase modulators input for increasing the OFC bandwidth. Since the signal at 26 GHz achieved a suitable electrical power, we have not used drivers, reducing the system complexity, cost, and overall power consumption. Sequentially, The analog transmission over fiber, or simply A-RoF, might experience fading effects induced by the fiber chromatic dispersion. This effect occurs due to the modulation sidebands beating in the photodetection process; as a result, a periodic power degradation can be observed depending on the RF frequency, fiber length, and dispersion^40^. Considering a A-RoF transmission over 12.5-km of standard SMF, fading nodes or null points are expected around 17.7, 30.6 and, 39.5 GHz. However, no fading nodes are noticeable due to the adjustments on the phase modulator bias voltages and mainly by the phase shifters, leading the entire optical frequency comb to sum in phase after the photodetection process.

**Figure 7:**
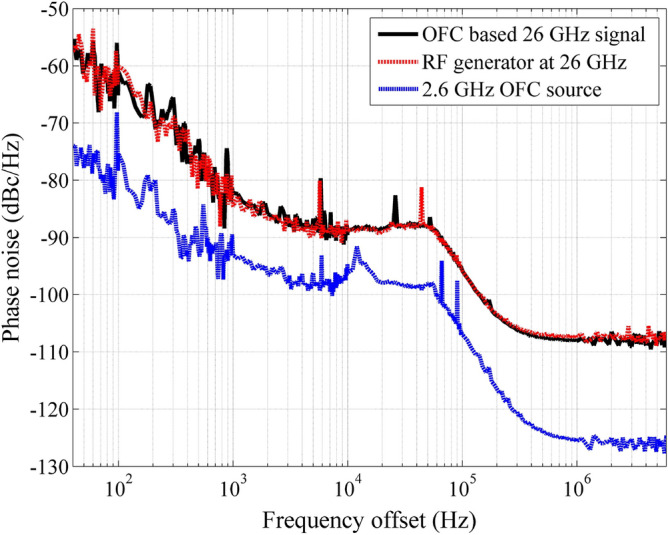
Phase noise measurements comparison among the 2.6 and 26 GHz OFC signals and th RF generator at 26 GHz.

In particular, we focused on using the tenfold multiplied electrical carrier (26 GHz), since this frequency range was standardized for high-throughput and low-latency 5G NR FR2 applications. Accordingly, it was necessary to use a BPF (4fv02-26000/t1000-k/k from K &L microwave) to isolate the high spectral purity mm-wave electrical carrier at 26 GHz, which represents the 5G NR up-converted signal, as displayed in Fig. 6b. The main filter features are 1-dB insertion loss and 1-GHz bandwidth centered at 26 GHz. Indeed, its use significantly increased the spectral purity in the entire frequency range, even for a 500 kHz span measurement, as presented in the figure inset. The electrical carrier presents suitable SNR and applicable electrical power of − 35 dBm without noticeable phase noise. In order to validate the OFC-based mm-wave signal generation, we have measured the 26 GHz carrier phase noise and properly compared it with the commercially available RF generator employed as OFC source, as reported in Fig. 7. The phase noise is a fundamental figure of merit of oscillators and photonics-based RF generators that are important to precisely characterize the harmonic stability, specially for critical applications, such as radars, communications at mm-waves, aerospace, and defense. The phase noise power spectrum for a multiplied signal based on external modulator can be described as^21^:

1 $$\begin{aligned} 10log_{10}(S(f)) = 10log_{10}(S_{res}(f)) + 10log_{10}(S_{e}(f) \cdot m^{^{2}}) \end{aligned}$$

where *m* is the frequency multiplication factor (FMF), $S_{res}(f)$ is the system residual phase noise, and $S_{e}(f)$ is the power spectrum of the RF driven signal. Therefore, the signal and system residual phase noise contribute to the resultant signal noise. In addition, the equation follows two distinct conditions: if $S_{res}(f) \ll S_{e}(f) \cdot m^{^{2}}$, the $10log_{10}(S_{res}(f))$ can be neglected and the resultant phase noise is approximately equal to the RF drive source phase noise added with $20log_{10}(m)$. The second condition occurs when $S_{e}(f) \cdot m^{^{2}} \ll S_{res}(f)$, as a consequence, the term $10log_{10}(S_{e}(f) \cdot m^{^{2}}$ is omitted and the total phase noise is based on the system residual phase noise. In particular, our system fulfills the first condition, as demonstrated in Fig. 7, which reports the phase noise measurement comparison among the 2.6 and 26 GHz generated waves and commercial RF generator at 26 GHz. For instance, the OFC RF source at 2.6 GHz obtained a phase noise of around − 75 dBc/Hz for a 50 Hz offset. As a result, the OFC-based tenfold multiplied signal (26 GHz) presented − 55 dBc/Hz for 50 Hz offset, following the $20log_{10}(10)$ increment on the entire range. Therefore, the proposed comb-based mm-waves generation remarkably does not increment the overall system noise, since it only depends on the multiplier factor. When compared with the commercial RF generator at 26 GHz, the OFC-based signal presents a very low phase noise, equivalent to the RF generator over the entire range, without adding phase distortions. As a conclusion, the proposed integrated OFC is capable of providing configurable RF multiplication up to mm-waves with notable phase noise feature, equivalent to the commercial generators used to generate its original OFC lines.

## Integrated OFC-based 5G NR system performance analysis

The next system implementation was to use the generated low phase noise 26 GHz carrier for the 5G wireless access network and evaluate its performance in real optical/wireless networks. Such signal was then amplified by an electrical amplifiers chain, namely: 12-dB gain and 4.5-dB noise figure (NF) amplifier (SBUA-400-12-010-k) from Fairview, followed by a 35-dB gain and 4.5-dB amplifier (QLW-18404540) from QuinStar. Afterwards, the 5G NR standard was incorporated to the 26 GHz carrier, using an AWG M9505A from Keysight and a mixer. The 5G NR configurations were based on the 3GPP conformance tests for FR2 user equipment (UE)^41^ with bandwidths up to 100 MHz. In particular, we have used numerology ($\mu$) = 2 for all bandwidths, giving rise to a 5G signal with 60 kHz subcarrier spacing (SCS). Specifically, the downlink shared channels (DL-SCH) have been adjusted for operating with multiple modulation schemes, aiming at 5G multi- applications. Considering the 100 MHz bandwidth signal, time slots from 1 to 35 have been modulated with 16-QAM (quadrature amplitude modulation), whereas the time slots from 41 to 75 have been occupied with 64-QAM modulation. In addition, we have used the time division duplexing (TDD) technique to maintain the time slots from 36 to 40 and from 76 to 80 set as uplink shared channels (UL-SCH). We have also configured the primary (PSS) and secondary synchronization signals (SSS), which are allocated to enable the user equipment to acquire the frame timing, as well as the cell identification.

**Figure 8:**
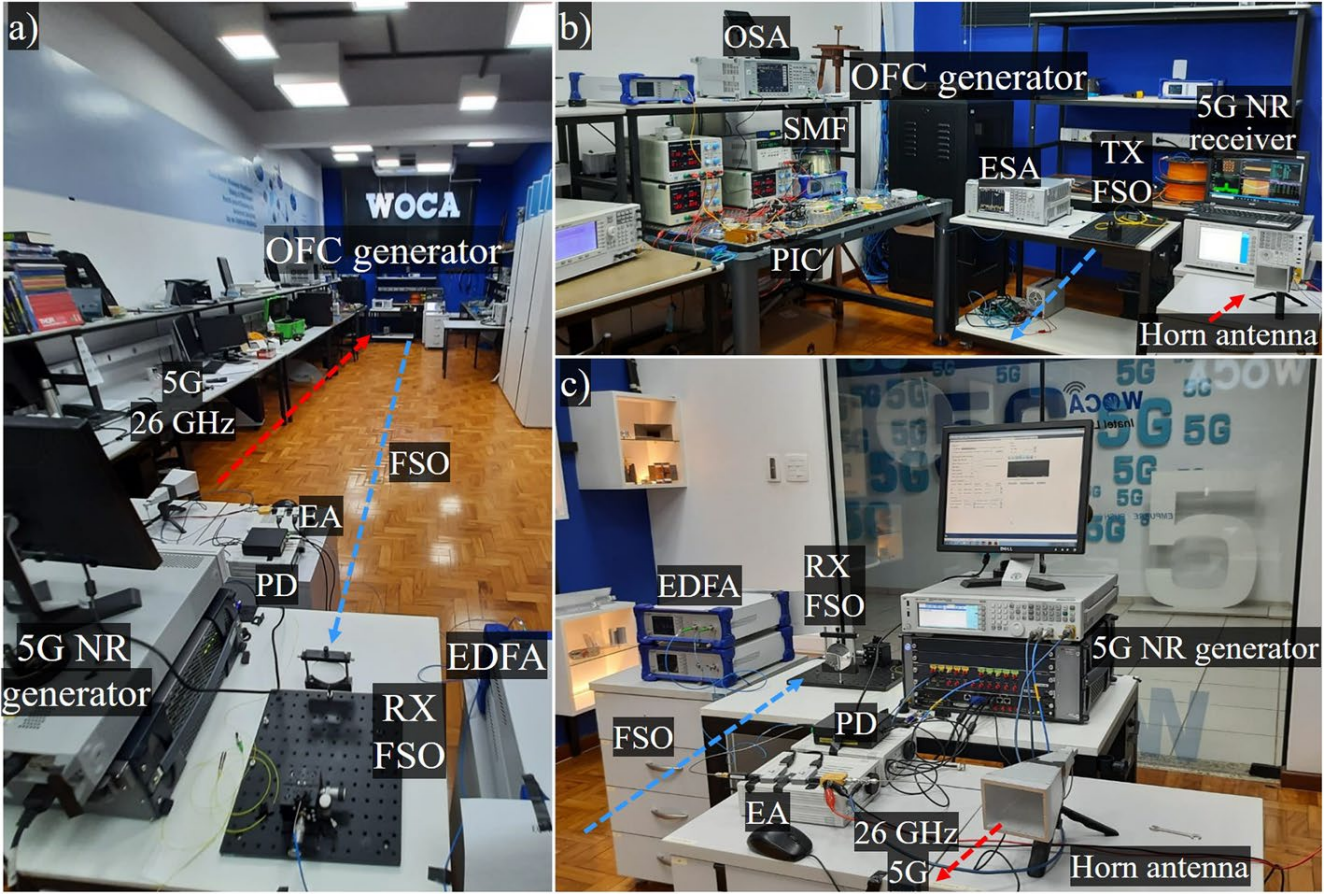
OFC-based 5G NR mm-waves system experimental setup photographs. (**a**,**c**) The back and front views, respectively, of the FSO reception, the PD followed by the electrical amplifiers chain, 5G NR generator and the TX horn antenna; (**b**) displays the PIC, optical link followed by the FSO transmitter, RX horn antenna and the 5G analyzer.

**Figure 9:**
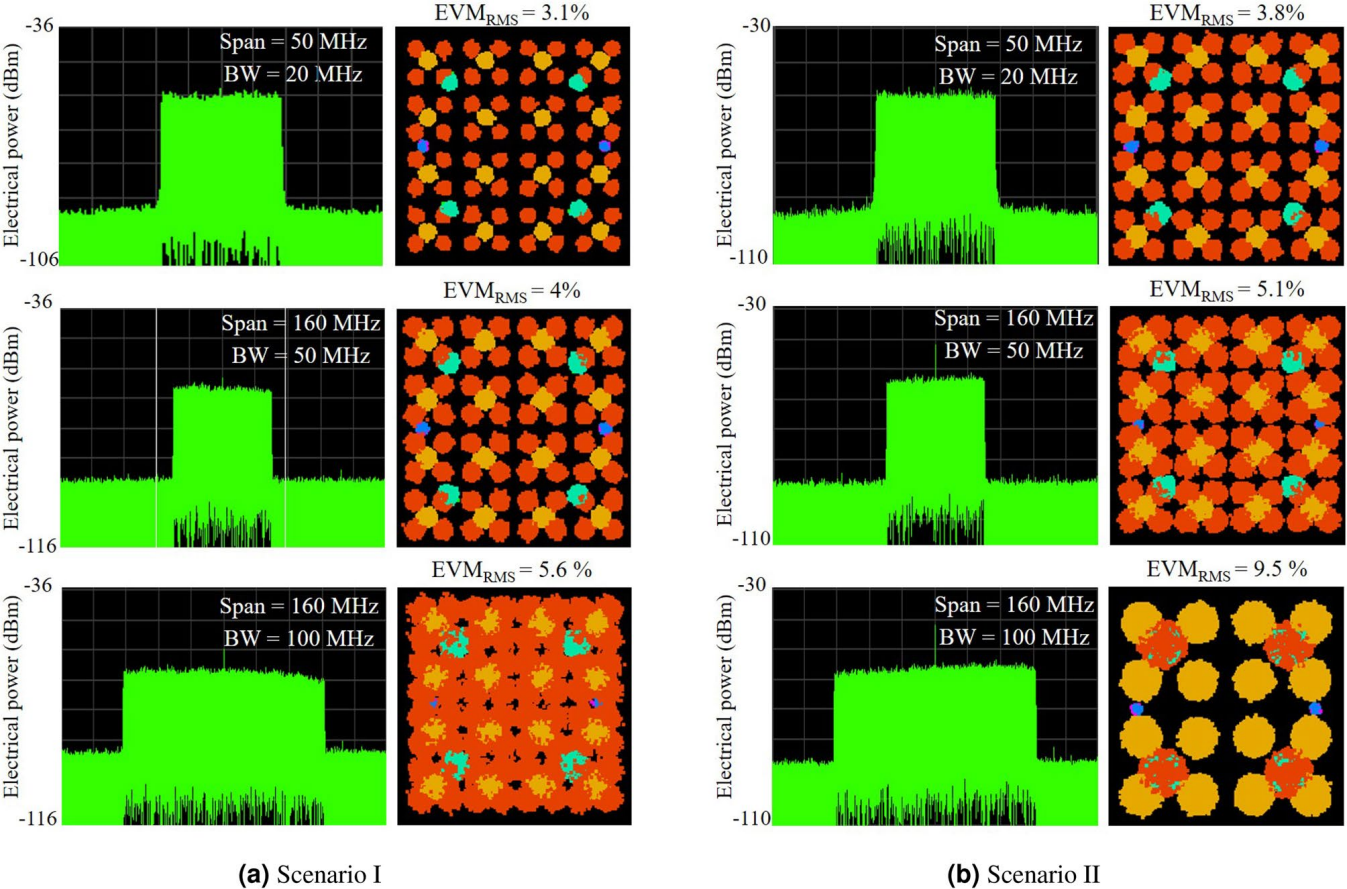
5G NR access points performance evaluation for the Scenarios I and II.

The up-converted 5G NR signal fed a 25-dBi gain horn antenna, enabling a 10-m reach wireless at 26 GHz. At the reception, an identical horn antenna has been used to capture the 5G signal that was amplified using a 35-dB gain low-noise amplifier (LNA). Afterwards, a VSA M9020A MXA from Keysight has been used to properly evaluate the 5G NR signals performance. The Release 15 focused on the eMBB scenario and introduced the EVM_RMS_ current requirements to support the 5G user equipment connection, namely: 17.5%, 12.5% and, 8.0% for QPSK (quadrature phase-shift keying), 16- and 64-QAM modulation schemes, respectively^41^. Figure 8 presents experimental setup photographs, encompassing the entire OFC-based 5G NR mm-waves system. Particularly, Fig. 8a,c show the back and front views of the FSO reception, respectively, including the photodetector followed by the electrical amplifiers chain, 5G NR generator, and transmitting horn antenna. Figure 8b displays the PIC, electrical cables mounted on an optical table, SMF link followed by the FSO transmitter, as well as the receiver antenna and 5G analyzer.

Figure 9a reports the 5G performance evaluation of the first scenario (Scenario I), i.e. OFC transmission over 12.5-km fronthaul to generate mm-waves and up-conversion of the 5G NR signal, enabling a 10-m wireless link. The indoor wireless environment is a research lab furnished with computers, pieces of equipment, and wood tables. The measurements have been performed during non-business hours, minimizing human interference and under line-of-sight (LOS) condition. As a first characterization, we have transmitted a 20-MHz 5G signal with 16- and 64-QAM modulations. The top of Fig. 9a presents the 20 MHz measured electrical spectrum at the reception and resulting constellations and EVM_RMS_. The measured electrical power was − 18 dBm with SNR higher than 30 dB. The EVM_RMS_ was only 3.1% with well-defined constellation symbols and no critical phase impairments, attaining 100 Mbits/s. Therefore, one can precisely distinguish the control and synchronism received symbols, denoted in blue and green on the constellation (BPSK and QPSK), as well as the data symbols in shades of orange (16- and 64-QAM). Sequentially, the signal bandwidth was increased to 50 MHz (250 Mbit/s). We have only reconfigured the 5G bandwidth, therefore, the channel power was kept around − 18 dBm, resulting in a slight SNR reduction. Nevertheless, The SNR decrement only impacted in a 1% EVM_RMS_ reduction. Finally, we have transmitted a 100 MHz bandwidth 5G signal aiming to increase the system throughput, as reported in the bottom of Fig. 9a. Despite the 100 MHz 5G signal wireless transmission at 26 GHz, the measured EVM_RMS_ was 5.6 %, fulfilling the 3GPP specifications with margin, i.e., 8% (64-QAM) and 12.5% (16-QAM). In this case, the OFC-based 5G NR hotspot provided 500 Mbit/s throughput.

In parallel, the split OFC has been transmitted throughout a 12.5-km optical midhaul followed by a 10-m FSO fronthaul and then a 10-m reach wireless access link at 26 GHz. The main obtained results from the second scenario (Scenario II) are reported in Fig. 9b, measured by the VSA. Figure 9b presents the spectra and resultant constellations for the measured 5G signals with 20-, 50- and 100-MHz bandwidth. One more time the channel power was around − 18 dBm, since we have used the same optical power at the photodetector input for both scenarios. Comparing with the scenario I (without FSO) and BWs of 20 MHz and 50 MHz, the obtained EVM_RMS_ has increased 0.7% and 1.1%, respectively, due to the additional EDFA used in the FSO link. Nevertheless, both BWs accomplished the 3GPP requirements with margins. For the 20 MHz signals, margins up 4.2% and 8.7% for 64-QAM and 16-QAM modulation, respectively, whereas the 50 MHz achieved up to 3% and 7.5% for 64-QAM and 16-QAM, respectively. As expected, the FSO followed by the wireless link impaired the received 100 MHz 5G NR signal in phase and magnitude, consequently, the measured EVM_RMS_ (9.5%) did not meet the specifications for 64-QAM. As an alternative, we have employed 16-QAM on the entire 100 MHz and also tested QPSK/16-QAM to overcome this performance drawback. Consequently, the hybrid PIC-based 5G NR mm-wave hotspot provided an $\hbox {EVM}_{\mathrm{RMS}}$ performance of 9.5%, fulfilling the requirements. In spite of the modulation index reduction, the system was able to provide up to 400 Mbit/s demonstrating the overall system feasibility, including the remarkable flexibility of FSO communications. Considering both scenarios, the total throughput of the proposed integrated OFC-based 5G NR system was 900 Mbit/s. The main 5G NR system experimental results are summarized in Table 2, as a function of scenario and bandwidth.

**Table 2:** The main 5G NR system experimental results.

Scenario	BW (MHz)	Modulation	$\hbox {EVM}_{\mathrm{RMS}}$ (%)	Throughput (Mbit/s)
Scenario I Fronthaul/Access	20	16-/64-QAM	3.1	100
	50	16-/64-QAM	4	250
	100	16-/64-QAM	5.6	500
Scenario II Midhaul/Fronthaul/Access	20	16-/64-QAM	3.8	100
	50	16-/64-QAM	5.1	250
	100	16-QAM QPSK/16-QAM	9.5	400
				300

## Conclusions

We have successfully proposed and implemented a flexible optical distribution network, using an integrated optical frequency comb as a low-phase noise RF generator operating in mm-waves and applied to 5G Xhauls. Particularly, our extremely-compact InP monolithically integrated OFC enables multiple applications in the 26 GHz band utilizing a single PIC, by taking advantage of the C-RAN architecture. It is based on cascaded phase modulators, making it broadly tunable in terms of operating wavelength and comb frequency spacing. Experimental results demonstrate an OFC spaced by 2.6 GHz with 27 optical tones at OSNR higher than 20 dB, which has been divided and distributed through two optical branches: a 12.5-km SMF fronthaul; a 12.5-km SMF midhaul, followed by a 10-m long FSO fronthaul. Posterior at the receiver side, high-speed photodetectors have been used to provide 2.6-GHz-separated in-phase electrical tones up to 41.6 GHz with suitable power and SNR. We focused on the 26 GHz generated carriers for simultaneously deploying two 5G NR 10-m reach wireless access networks operating in FR2 with up to 100 MHz bandwidth. Those carriers provided remarkable low phase noise levels as low as − 55 dBc/Hz for a 50 Hz offset, which is equivalent to that of the RF generator. Both sub-systems have been evaluated in accordance to the 3GPP Release 15 requirements in terms of EVM_RMS_ for different modulations. Overall, the proposed optical-wireless system achieved 900 Mbit/s in accordance to 3GPP, demonstrating the PIC feasibility to generate tunable low-phase-noise mm-waves signals. Future works regard the PIC use to generate even higher frequencies, as well as integrating our system with 6G enabler technologies, including artificial intelligence, machine learning, and visible light communications for the access networks.

## Data Availability

The datasets generated/analyzed during the current experimental study are available from the corresponding author on a reasonable request.
